# Cancer vulnerability in an indigenous Himalayan population in Arunachal Pradesh

**DOI:** 10.3332/ecancer.2022.1405

**Published:** 2022-05-30

**Authors:** Chaitan Kumar, Moirangthem Momocha Singh

**Affiliations:** Department of Management & Humanities, National Institute of Technology, Itanagar, Arunachal Pradesh, India; a https://orcid.org/0000-0002-5117-1083; b https://orcid.org/0000-0002-2507-698X

**Keywords:** cancer, knowledge, attitude, practice, prevention, indigenous population

## Abstract

Cancer incidence and its related mortality has been a public health concern for Arunachal Pradesh in India. However, there is a lack of evidence about the knowledge, attitude and practice (KAP) for cancer risk factors, screening programmes and preventive behaviour – especially among indigenous tribal populations. A cross-sectional survey was conducted using Google Forms from 16 September 2020 to 2 January 2021 among an indigenous population of Arunachal Pradesh. Snowball sampling was used to enrol 565 participants aged ≥18 years (264 were male and 301 were female). Univariate and bivariate analyses were conducted using SPSS version 23 to test the hypothesis of KAP. (There is no difference in the level of knowledge / in the attitude / level of practice among study participants with respect to any independent (socio-demographic and other) factors.) The Papumpare Cancer Registry reported the highest cancer density among women and the second highest among men among all population-based cancer registries in India (Indian Council of Medical Research, Report of National Cancer Registry Programme, Bengaluru, India 2020). Knowledge about the cause of cancer and risk factors was poor among 23% of the respondents. Attitude towards screening was negative among 14.9%. Practice levels to prevent cancer were also low (31%). More than 50% of the cases were treated outside the state and at private hospitals. Knowledge about cancer symptoms and risk factors was limited in the population. There is a need for more effective health promotional services in the state. Mass screening facilities and behavioural change activities are required and could be disseminated through social media platforms. Our analysis of a north-eastern region of India, which has unique geographical and cultural characteristics, informs future policy designs and other related studies for controlling cancer in the area.

## Background

**Figure 1.**
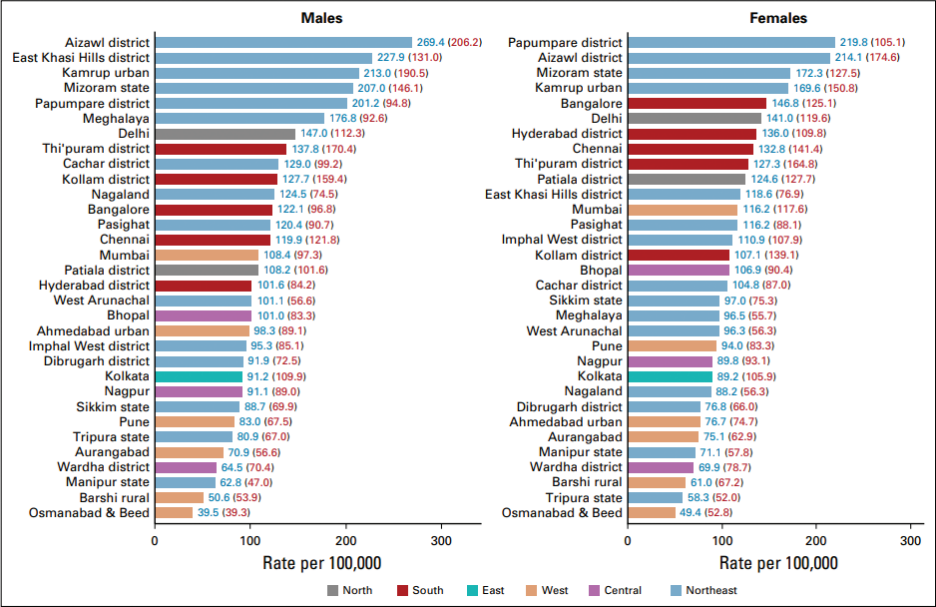
Geographical distribution of cancer rate per 100,000 people in India. Source: Report of National Cancer Registry Programme (ICMR-NCDIR), Bengaluru, India 2020.

Cancer poses a major public health problem worldwide owing to its high prevalence and incidence along with the associated socio-economic burden. The projected number of patients with cancer in India was 1,392,179 in 2020, with a higher reported incidence among women (712,758) than men (679,421) [1]. The top five common cancer-reported sites are breast, lungs, mouth, cervix uteri and tongue. As shown in Figure 1, there is marked heterogeneity in the cancer incidence (age-adjusted rate per 100,000 people) among both men and women across different regions within Indian states [1].

The higher incidence of cancer was attributed to higher consumption of tobacco in the North-eastern states, followed by the West and Central regions of India [7]. Among men, the most common cancer sites included lungs, mouth, oesophagus and stomach. Among women, the predominant cancer site was breast, followed by cervix uteri and ovaries [7].

Most of the knowledge, attitude and practice (KAP) studies have been conducted among the general population of India or in different states like Chennai, Kerala, Punjab and Kashmir. These studies documented low levels of knowledge about cancer risk factors, the importance of screening and early detection and protective behaviours [2–4]. An individual’s level of practice of healthy behaviours is even lower than his/her knowledge scores, and there is limited awareness of risk factors among the North-eastern general population [5].

In the extant literature, there is no evidence related to KAP about cancer and its risk factors, screening programmes and protective behaviours among indigenous tribes of Arunachal Pradesh in the North-eastern states of India.

## Objectives

- To assess the knowledge about cancer risk factors and preventive behaviours among an indigenous population.
- To explore the indigenous population’s awareness of screening methods.
- To determine the socio-economic impact of cancer on families.
- To determine the perception of public for stakeholder’s role in the population.

## Methods

This online cross-sectional survey was conducted using Google Forms from 16 September 2020 to 2 January 2021 among an indigenous population of Arunachal Pradesh.

### Brief geographical characteristics of the North-eastern states of India and Arunachal Pradesh

Indian has 28 states and 8 union territories. The north-eastern part of India consists of eight states. Most of the population (45,587,982) belong to the hilly and tribal population, per the 2011 Census [6]. Arunachal Pradesh comprises 3% (1,382,611) of the total population of the North-eastern states of India (68.8% tribal population) [6]. The state has a sex ratio that is 938. The literacy rates among women and men are 57.7% and 72%, respectively [6]. The five major tribal communities—Monpa, Apatani, Nissi, Adi, and Galo—constitute 41% (558,546 people) of the population (Table 1).

### Cancer epidemiology in Arunachal Pradesh

Cancer registries are maintained for three major geographical sites of Arunachal Pradesh (Table 2): Papumpare (central region), Pasighat (eastern region) and West Arunachal (western region of Arunachal Pradesh). Approximately 3017 cancer cases were reported in Arunachal Pradesh from 2014 to 2016 (51% men and 49% women) [7]. The age-adjusted rate of cancer is highest in Papumapre region, for both men and women [7].

The top four cancer sites among men in West Arunachal Pradesh are stomach, liver, oesophagus and lungs. Among women, stomach, breast, cervix uteri, thyroid and liver are the most prevalent in West Arunachal [7]. In Pasighat, the proportion of stomach cancer (18.1%) was highest among men, followed by lung (7.8%) and liver (5.9%) cancer. In women, the cervix uteri was the leading cancer site (18.5%), followed by breast (16.8%) and stomach (9.6%) cancer [7].

The state has 1 medical college, 6 general hospitals, 15 district hospitals, 51 community healthcare centres, 148 primary healthcare centres, 4 urban primary healthcare centres and 582 sub-healthcare centres [8]. Arunachal Pradesh has developed 136 health and wellness centres, which provide non-communicable disease (NCD) screening facilities along with screening of 3 types of cancers, breast, cervical and oral, for those aged ≥30 years [9]. A tertiary cancer care facility was established under the new medical college; however, a dedicated cancer hospital is absent in the state [9].

### Study population and sampling method

Arunachal Pradesh has a population of approximately 1.4 million. Owing to the COVID-19 pandemic and travel restrictions, the survey was conducted online. We used Google Forms via online platforms in urban areas where most people have Internet access. Therefore, we selected districts where the urban population of the indigenous tribes was predominant. Hence, we adopted convenience sampling for selecting the districts.

Furthermore, snowball sampling was used to enrol the participants. We enrolled 565 adults (aged ≥18 years) belonging to a tribal population and residing in urban areas. We requested our relatives and colleagues to forward the link to the questionnaire through email and WhatsApp. Respondents who were aged <18 years, lived outside the state and had not heard about cancer were excluded from the analysis.

We developed and pre-tested the questionnaire by reviewing the existing literature and seeking expert guidance from faculties of the University of Delhi. Validated questions from similar questionnaires on KAP in India were utilised [2–5]. The content and face validity of the tool was established by five subject experts. The tool was pre-tested with 33 participants, and test–retest reliability was established. The overall Cronbach’s alpha value exceeded 0.85, indicating good internal consistency.

Participants were assured of the confidentiality of their personal information. Informed consent was obtained from each participant with a statement that data would be used for academic and research purposes only. Ethical permission was granted by the institute’s research committee before conducting this study. An approval letter from the Directorate of Health Services of Arunachal Pradesh was also sought.

### Statistical analysis

The data were cleaned in Excel and exported in the IBM SPSS version 23. The data analysis included univariate, bivariate and multivariable regression modelling. The socio-demographic variables and KAP parameters about cancer risk factors and prevention were analysed and are presented using frequency and percentage.

The variables representing knowledge about symptoms and causes of cancer (multiple response variables), as well as about the ability of vaccines to prevent certain cancers were utilised to generate a variable representing total knowledge score. Each correct response was scored 1 and the response of ‘no knowledge’ was coded 0. The total knowledge score ranged from 0 to 22. A categorical variable representing cancer/NCD patients being at high risk for COVID-19 infection (mutually exclusive choices) was also generated (poor: <33%; moderate: 33–65%; good: ≥66%).

The variables representing attitude included awareness about a screening programme, opinion about self-assessment knowledge in helping early screening of cancer and risk reduction, opinions about telemedicine advice to improve the screening and treatment, and preference of screening for cancer at one’s doorstep to save money. The responses to these were mutually exclusive choices. Each response of ‘yes’ was scored 1, and each response of ‘no’ was scored 0. The total score ranged from 0 to 4. Binary categorical variables representing the attitude (positive: 1 and negative: 0) was also generated.

The variables representing practices included steps taken to reduce the risk of cancer (multiple responses variables), visiting a hospital for a preventive check-up, online searches regarding cancer-related services and information, and participation in a cancer screening programme (mutually exclusive choices). Each correct response as well as response of ‘yes’ was scored 1 and each response of ‘no’ was scored 0. The total score ranged from 0 to 14. A categorical variable representing the three levels of practice (low: <33%; moderate: 33–65%; high: ≥66%) was also generated.

A bivariate analysis was conducted to explore the factors affecting KAP about the prevention of cancer. Chi-square tests were also performed. A binomial regression model was tested for factors that were significantly associated with the nature of attitude about cancer prevention. Similarly, multinomial regression models were tested for factors that were significantly associated with the level of knowledge and practices about cancer prevention. P-values < .05 were deemed significant.

## Results

### Demographics

There were 565 participants, aged 18–68 years. The mean age was 27.9 ± 7.8 years. Most of them were aged 18–34 years, women, well-educated, single and unemployed at the time of the survey. The Arunachali community constituted 92% of the study sample (n = 520), and most belonged to the schedule caste and tribe community (n = 530). Most did not own a ration card (for getting subsidised rations) and did not have any kind of health insurance scheme (Table 3).

### Knowledge about cancer among participants

#### Sources of information about cancer

Healthcare workers were the main source of knowledge about cancer for nearly all participants. Other popular options were schools/colleges, social media and print media (Table 4).

#### Knowledge about the causes of cancer

Nearly all participants mentioned the use of tobacco products to be the cause of cancer, and three-quarters mentioned second-hand smoke to be one of the causes of cancer. Alcohol consumption was considered to be carcinogenic by 74% of the participants. The other causes of cancer mentioned by participants included exposure to air pollution and indoor smoke from solid fuels, eating processed foods and the use of areca nut/supari or betel. The other causes of cancer mentioned by participants included exposure to harmful ultraviolet rays, lack of physical activity, viral infections, high body mass index and consumption of sugary drinks (Table 4).

#### Knowledge about the symptoms of cancer

The most perceived cancer symptoms were a lump in the breast, ulcers/patch/growth in mouth that did not heal for more than 2 weeks, difficulty in chewing or swallowing and change in shape or size of breast and pain (Table 4). Furthermore, 70% of the participants had not heard about cancer screening services available at government hospitals or health and wellness centres, and most of them were not aware about the role of vaccines in preventing certain cancers. Two-fifths agreed that the COVID-19 pandemic made patients with cancer or other NCDs more vulnerable.

Approximately half had information on cancer from three to four sources, and more than half were aware of five to eight causes of cancer and more than three symptoms of developing cancer. In sum, the level of knowledge was poor among 23.7% (*n* = 134), moderate among 41.4% (*n* = 234) and good among 34.9% (*n* = 197) of the participants. Table 4 provides the major knowledge sources, the perceived cancer causes and the perceived symptoms of cancer, respectively.

#### Practices of cancer prevention

Concerning preventive measures taken, tobacco and alcohol use were avoided by most participants. Other common measures included avoiding areca nuts and processed food. Engagement in daily physical activity and maintaining a healthy weight were also common. Nearly 6.6% mentioned that they did not use any preventive measure (Table 5).

Most had not visited the hospital for a preventive check-up. Of those who went, most were informed by their physicians to avoid exposure to the risk factors.

Only 5.8% had participated in a cancer-specific screening programme. Of these, 60.6% availed the service free from the government facility, whereas 15.2% paid for the service at a private facility. The reasons behind choosing a private facility were unavailability of screening at the nearest government hospital or that it was too far.

Only 11.7% had participated in a screening programme for other NCDs. Of those, 30.3% had paid more than 5,000 rupees (INR). More than half had searched online about cancer risk reduction/symptoms/nutrition/other services.

**Figure 2.**
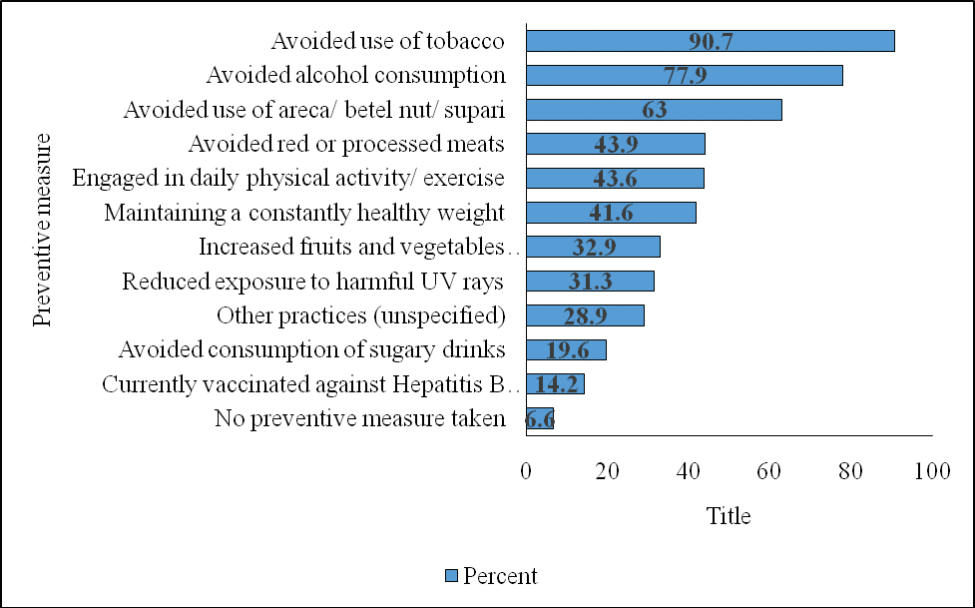
Distribution of preventive measures against cancer taken by participants (multiple responses permitted).

The assessment of indicators of practice revealed that the distribution of the levels of practice about cancer appropriate behaviour was similar across three categories, including low, medium and high. Majority of the respondents (90.7%) wished to quit tobacco. Figure 2 shows the participants’ responses concerning preventive measures.

### Attitude towards cancer prevention

#### Attitude about getting screened for cancer and other NCDs

When asked, 5.8% mentioned that the risk factors were not serious, while 29.9% mentioned that they were planning for future screening. Nearly two-thirds were not willing to explain their reason. Most mentioned that they would prefer screening facility at healthcare centres. Most opined that self-assessment knowledge will help in early screening of cancer and promote risk reduction. Most also said that cancer is a high public health concern in Arunachal Pradesh (Table 6).

#### Family history of cancer and its financial implications

Ninety-seven responded stated that they had some family history of cancer, and the treatment costs are provided in Table 7. Nearly one-third mentioned job loss owing to cancer treatment as a chief financial burden. Some participants had to borrow money or sell property/jewellery to bear the costs (Table 8).

#### Perceived role of government in controlling cancer and other NCDs

**Figure 3.**
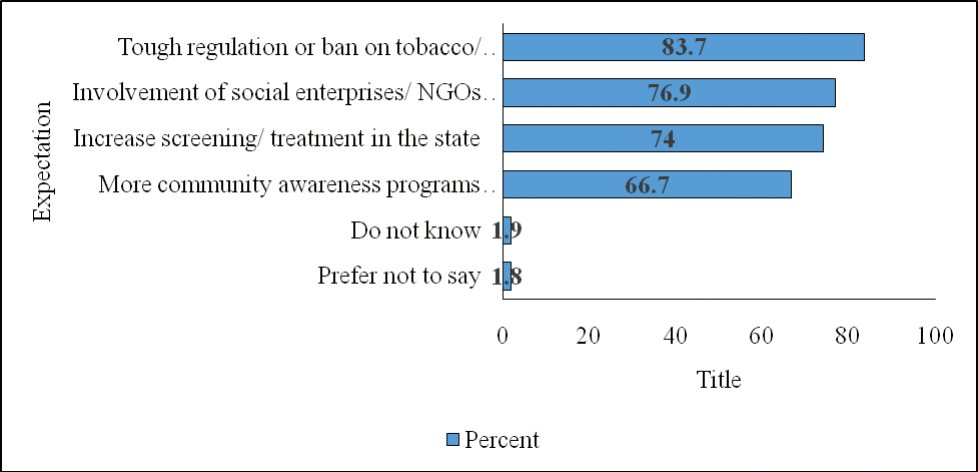
Expectations from the government and other stakeholders towards control of cancer and other NCDs (multiple responses permitted).

Respondents believe that the government and other stakeholders have crucial roles to play. When asked about their expectation from the government and other stakeholders towards cancer control, three-quarters mentioned that the screening/treatment services in the state must be increased. Two-thirds suggested that more community awareness programmes should be conducted. However, most mentioned that there must be tough bans on the use of tobacco, alcohol and other cancer-causing products. Furthermore, most mentioned the need to involve social enterprises or non-governmental organisations to obtain affordable prices (Figure 3).

### Factors affecting KAP

#### Factors affecting knowledge

In the bivariate analysis, the outcome variables were identified as per the study’s objectives, i.e., KAP about cancer prevention. The factors influencing these outcome variables, also known as independent variables, were tested using chi-square tests as all outcome variables were categorical. Following the bivariate analysis of each outcome variable, the significant associations (*p* < 0.05) were tested using regression models. Beginning from Tables 9 to 11, each bivariate analysis was conducted using a chi-square test, followed by a regression model (binomial or multinomial). The purpose of regression modelling was to control for confounding factors and thus identify the factors that truly affect the level of knowledge independent of each other. The null hypothesis was as follows: There is no difference in the level of knowledge among participants with respect to any independent (socio-demographic and other) factors.

As shown in Table 9, participants’ age, education, marital status, religion, number of cancer information sources, number of known causes of cancer, number of known causes of symptoms and number of preventive measures taken were significantly associated with the level of knowledge about cancer.

Better knowledge about cancer was more prevalent in younger age groups as compared to their older counterparts. Similarly, the prevalence of a moderate or good level of knowledge was higher among Christians, those who were more educated, and individuals with a moderate knowledge about symptoms, causes or preventive measures as compared to their counterparts.

#### Factors affecting attitude about cancer

The factors affecting the attitude were tested using chi-square tests of significance, followed by a binomial logistic regression model as the outcome variable on attitude had two categories. The null hypothesis was as follows: There is no difference in the attitude about cancer among participants with respect to any independent (socio-demographic and other) factors.

As shown in Table 10, the proportion of negative attitude was higher among men (61.9%) and positive attitude was higher among women (55.9%) (*p* < 0.05). Furthermore, a higher proportion of individuals with knowledge about fewer (up to 4) causes of cancer held more negative attitudes than did their counterparts. Positive attitude was significantly associated with an increase in the number of known causes of cancer, increase in the number of known symptoms and increase in the number of preventive measures taken by participants.

#### Factors affecting practice about cancer prevention

The factors affecting the level of practice were tested using chi-square tests of significance, followed by a multinomial logistic regression model, as the outcome variable on the practice level had more than two categories. The null hypothesis was as follows: There is no difference in the level of practice for cancer prevention among participants with respect to any independent (socio-demographic and other) factors.

**Figure 4.**
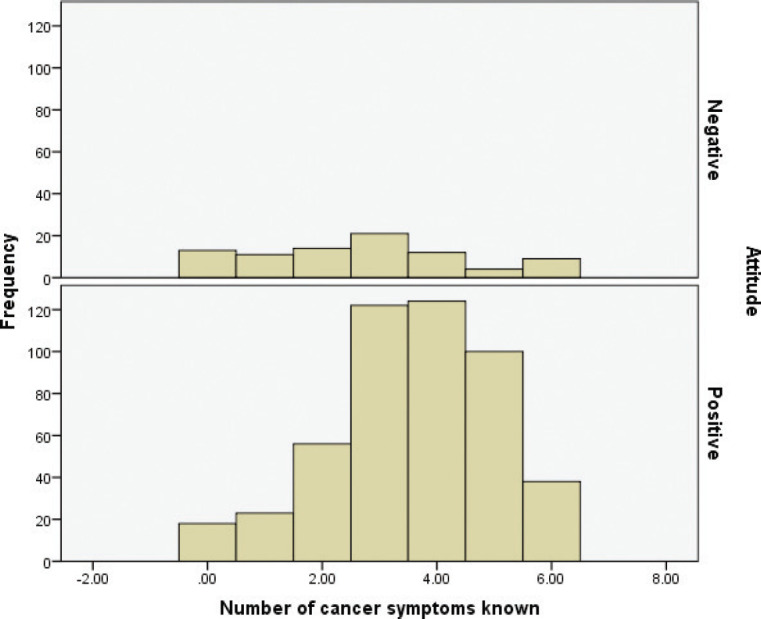
Knowledge about cancer symptoms and attitude towards cancer prevention.

As shown in Table 11, the youngest age group (18–24 years) practiced a high level of cancer prevention, while those aged 25–34 years practiced a moderate level (*p* < 0.05). Similarly, a low level of practice about cancer prevention was more common among those who were less educated than their counterparts (*p* < 0.05). Any level of practice of cancer prevention was much higher among those who were single or Christians (*p* < 0.05). A moderate to high level of practice was found among individuals with no ration card, no insurance scheme, those who obtained information from three to four sources, who knew five to eight causes of cancer, who knew more than three symptoms and who knew the risks associated with NCDs and COVID-19 (*p* < 0.05). The relationship between the knowledge of symptoms and prevention attitude is shown in Figure 4.

## Discussion

Only 35% of the participants had good knowledge, followed by 40% with a moderate level of knowledge, on various aspects of cancer. People knew that tobacco and alcohol consumption were cancer-causing risk factors, which has been reported in the Indian Council of Medical Research (ICMR) reports [7]. Most believed that avoiding alcohol and tobacco can reduce vulnerability. Awareness of common cancer symptoms is limited to all in the community, which is similar to the other states in the North-eastern region in India [5]. One study in Assam found that 92% of the participants enjoyed chewing areca nuts and only 18% tried to quit [10]. However, in our study, approximately 63% of the indigenous population had quit areca nut chewing owing to high levels of awareness in the community.

Healthcare workers were the participants’ main source of information. Religious places were rarely noted as generating awareness about cancer. This is an important finding because in India, most people are religious and adhere to the teachings imparted at such places. Thus, to improve awareness and health-promotion activities, religious institutions should disseminate cancer-related information.

Participants’ KAP concerning NCD screening was very low. Most had never heard of the Ayushman Bharat Health and Wellness Centre for NCD screening. Very few had receiving screening. This coincided with prior results which showed that in the North-eastern state of Assam 92.9% of the participants were not aware of screening or had not been screened [11].

This study looked at the available literature on cancer awareness, attitudes and screening practices among the population in the north-eastern part of India. A variety of variables contributed to low cancer screening adherence: a lack of understanding, knowledge and practice; low levels of psychological threat; delayed symptoms and signs in the early stages; stigmatisation linked to cancer; anxieties; expenditures; household duties; and humiliation. In this study, most participants knew that tobacco was associated with a high risk of cancer. In the research carried out in emerging and impoverished states, similar outcomes were found [11]. Nevertheless, these findings contrast with those of the research involving people attending the obstetrics and gynaecology department of a public health facility [4]. Despite adoption of the National Programme for Prevention and Control of Cancer, Diabetes, Cardiovascular Diseases and Stroke in the state, the population has lesser awareness of cancer screening [9]. This could be because primary healthcare clinics have limited facilities for cancer screening.

During our assessment, we found that most participants were aware of the HPV vaccine, but only a handful had taken it. Women, in another study, felt that early screening and HPV vaccination might boost their immune system, even though most women had never been screened.

There was a significant difference in the KAP among participants with respect to various socio-demographic and other factors. Age, education, marital status, religion and number of sources of knowledge were independent predictors of knowledge. Other studies also found that knowledge and practice had significant associations with the level of education and family income [12]. Education, age and per capita income were independent predictors of information, attitudes and practice of cancer screening, which also mirrored prior research globally [13].

To date, no study has explored the KAP of indigenous tribes concerning cancer risk factors, screening behaviours, adoption of healthy lifestyles and availability of various health facilities in Arunachal Pradesh. Our unique findings thus inform policy level decisions to curb cancer in this area.

### Limitations and future scope

A key limitation of this study is that respondents completed online questionnaires and were recruited only from urban areas. Future studies should seek different populations to generalise the results.

## Conclusion

KAP concerning cancer risk was significantly related to participants’ health- and demographic-related characteristics. The risk factors of cancer in Arunachal Pradesh are high. Public and private providers have a good opportunity to serve the people. Screening and treatment must be improved in the state. Dedicated cancer institutes, diagnosis facilities, sufficient human resources and palliative care centres are required. Improved telemedicine or extending national cancer grid service can be attached with the Tomo Riba Institute of Health And Medical Sciences for better patient services. The state-specific preventive plan should focus on the local ethnic concern of various risk factors like food habits. Knowledge and behavioural change strategies concerning the availability of affordable services should be a public health agenda. Village-wise screening camps could be utilised for mass screening among eligible populations. To overcome the resource constraints, private players or non-profit partners with affordable services should be utilised so patients do not have to migrate to other states for diagnosis and treatment. Knowledge about the relevant government schemes should also be disseminated. Regulations related to tobacco and alcohol need to be tightened. Increasing the number of health workers who work in cancer screening at all hospitals could also further increase detection rates. Self-assessment tools and telemedicine services can play important roles in risk reduction and early detection. Lastly, studies related to the food and culture of Arunachal Pradesh should be carried out to elucidate other factors associated with cancer in the state.

## List of abbreviations

APL: Above poverty line; BPL: Below poverty line; Govt.: government; HPV: Human papillomavirus; ICMR: Indian Council of Medical Research; KAP: Knowledge, attitude and practices; NCD: Non-communicable disease; NCDIR: National Centre for Disease Informatics and Research; Rs: Rupee; SC: Scheduled caste; ST: Scheduled tribe

## Conflicts of interest

The authors declare that they have no conflicts of interest.

## Authors' contributions

The corresponding author was involved in the study’s conception, design, collection and analysis of data and drafting the report. The second author assisted in all stages and made suggestions for technical writing. Both authors approve the submission of this manuscript and are accountable for all aspects of the work.

## Funding

This research work was not funded by any organisation.

## Figures and Tables

**Table 1.** Composition of a major indigenous population in Arunachal Pradesh.

S. No	Tribe	Population	%
1	Monpa	12,398	0.92
2	Apatani	44,353	3.30
3	Nissi	286,770	21.31
4	Adi/Adi miniyuong/Miniyung	118,477	8.80
5	Talgalo/Adi Gallong/Gallong	96,548	7.17
	**Total**	**558,546**	41.50

**Table 2.** Cancer incident rate in various registries.

Registry	Age-adjusted rate		Crude rate		Possibility of cancer in lifetime	
	Women	Men	Women	Men	Women	Men
Papumpare	219.8	201.2	105.1	94.8	1 in 4	1 in 4
Pasighat	116.2	120.4	88.1	90.7	1 in 8	1 in 7
West Arunachal	96.3	101.1	56.3	56.6	1 in 10	1 in 8

Source: Report of National Cancer Registry Programme (ICMR-NCDIR), Bengaluru, India 2020

**Table 3.** Socio-demographic characteristics of the participants (*N* = 565).

Characteristic	Category	*n*	%
Age (years)	18 to 24	237	41.9
	25 to 34	228	40.4
	35 or older	100	17.7
Sex	Women	301	53.3
	Men	264	46.7
Education	Up to higher secondary	173	30.6
	Graduate	310	54.9
	Post-graduate and above	82	14.5
Marital status	Single	434	76.8
	Married	131	23.2
Religion	Christian	264	46.7
	Hindu and others	200	35*.4*
	Buddhist	101	17.9
Community	Arunachali	520	92.0
	Non-Arunachali	45	8.0
Tribe of Arunachali Community	Nyishi	141	27.1
	Monpa	90	17.3
	Apatani	86	16.5
	Adi	66	12.7
	Galo	65	12.5
	Tagin	58	11.2
	Nocte	5	1.0
	Khampti	2	0.4
	Tangsa	2	0.4
	Deori	1	0.2
	Memba	1	0.2
	Mishmi	1	0.2
	Sajolang	1	0.2
	Tai Khamyang	1	0.2
	Tutsa	1	0.2
Social caste	Scheduled caste (SC)/ Scheduled tribes (ST)	530	93.8
	Non-SC/ST	35	6.2
Employment	Non-working (homemaker/retired/unemployed/student)	343	60.7
	Govt./non-govt. employee	130	23.0
	Daily wage labourer/self-employed	92	16.3
Type of ration card	Do not have	371	65.7
	Below Poverty Line (BPL)	119	21.1
	Above Poverty Line (APL)	75	13.3
Type of health insurance availed	Govt./private	56	9.9
	No insurance	509	90.1

**Table 4.** Knowledge about cancer among the participants (*N* = 565).

Parameter	Category	*n*	%
Sources of knowledge about cancer (multiple responses permitted)	Healthcare workers	563	99.6
	School/college (books or events)	438	77.5
	Social media (Facebook/WhatsApp/YouTube)	429	75.9
	Electronic media/print media	380	67.3
	Any other source (peer learning/relatives, parents/friends, non-governmental organization)	173	30.6
	Religious institutions	46	8.1
Knowledge about causes of cancer (multiple responses permitted)	Use of tobacco products	554	98.1
	Exposure to tobacco smoke from others	438	77.6
	Alcohol consumption	418	74
	Exposure to air pollution and indoor smoke from solid fuels	309	54.7
	Eating processed foods	275	48.7
	Areca/betel nut/supari use	235	41.6
	Skin exposure to harmful ultraviolet rays	213	37.7
	Lack of physical activity	211	37.4
	Viral infections	195	34.5
	Other reasons (unspecified)	175	31
	High body mass index (being overweight)	169	29.9
	Low fruit and vegetable intake	56	9.9
	Drinking sugary drinks	52	9.2
Perceived knowledge about symptoms of cancer (multiple responses permitted)	Lump in breast	486	86.1
	Ulcers/patch/growth in mouth which did not heal for more than two weeks	426	75.4
	Difficulty in opening mouth chewing or swelling	398	70.4
	Change in shape and size of breast and pain	362	64.1
	Bleeding between periods	185	32.7
	Others (not mentioned here but know through medical reports about other symptoms)	139	24.6
	Bleeding after menopause	92	16.3
	Unaware of any symptom	54	9.5
Heard about screening services at government hospitals or HWCs	Yes	117	20.7
	No	396	70.1
	Do not want to answer	52	9.2
Whether Hepatitis B or HPV vaccine can prevent cancer	Yes	83	14.7
	No	413	73.1
	Do not want to answer	69	12.2
Whether people with cancer and other NCDs are more vulnerable during the COVID-19 pandemic	Yes	244	43.2
	No	268	47.4
	Do not want to answer	53	9.4
Whether any symptoms/risk factors are associated with you	Yes	35	6.2
	No	530	93.8
Level of knowledge	Poor	134	23.7
	Moderate	234	41.4
	Good	197	34.9
Number of sources of information about cancer	Up to two sources	139	24.6
	Three to four sources	303	53.6
	More than four sources	123	21.8
Number of causes of cancer known	Up to four causes	184	32.6
	Five–eight causes	329	58.2
	More than nine causes	52	9.2
Number of symptoms of cancer known	Up to three symptoms	278	49.2
	More than three symptoms	287	50.8

**Table 5.** Practice about cancer prevention among the participants (*N* = 565).

Parameter	Category	*n*	%
Preventive measures taken (multiple responses permitted)	Avoided use of tobacco	512	90.7
	Avoided alcohol consumption	440	77.9
	Avoided use of areca/betel nut/supari	356	63
	Avoided red or processed meats	248	43.9
	Engaged in daily physical activity/exercise	246	43.6
	Maintaining a constantly healthy weight	235	41.6
	Increased consumption of fruits and vegetables	186	32.9
	Reduced exposure to harmful ultraviolet rays	117	31.3
	Other practices (unspecified)	163	28.9
	Avoided consumption of sugary drinks	111	19.6
	Currently vaccinated against Hepatitis B and/or HPV	80	14.2
	No preventive measure taken	37	6.6
Ever visited hospital for any preventive check-up	Not visited yet	450	79.6
	Visited	115^a^	20.4
Whether doctor told to quit exposure to risk factors (*n* = 115)	Yes	85	73.9
	No	30	26.1
Whether participated in any cancer specific screening/diagnosis programme	Yes	33^b^	5.8
	No	532	94.2
Place of receiving screening services (*n* = 33)	Free at govt. facility	20	60.6
	Both private and govt.	7	21.2
	Paid private	5	15.2
	At private with health insurance coverage /cost	1	3.0
Reason for choosing private facility (*n* = 6)	Screening not available at nearest government hospital	4	66.7
	Government centre far away	2	33.3
Went for screening for any other NCD	Yes	66	11.7
	No	499	88.3
Expenditures incurred on screening (*n* = 66)	Zero	12	18.2
	Below rupee (Rs) 500	8	14.8
	Rs 500 to 1,000	12	18.2
	Rs 1,000 to 5,000	14	21.2
	Rs Above Rs 5,000	20	30.3
Ever searched online for cancer risk reduction/symptoms/nutrition/other services for patients with cancer	Yes	320	56.6
	No	245	43.4
Whether self or family members tried any traditional methods of cancer prevention	Yes	42	7.4
	No	523	92.6
Number of preventive measures practiced	Up to three	156	27.6
	Four–six	276	48.8
	More than six	133	23.5
Practice level	Low	177	31.3
	Moderate	191	33.8
	High	197	34.9

**Table 6.** Attitude about screening for NCDs among the participants (*N* = 565).

Parameter	Category	*n*	%
Causes for not getting screened (*n* = 499)	Risk factors are not serious	29	5.8
	Planning for future screening	149	29.9
	Do not want to explain	321	64.3
Whether prefer to be screened for NCDs at one’s doorstep in the future to save money	Will prefer at health centres, not at home	363	64.2
	Yes, with affordable paid services	202	35.8
Whether self-assessment knowledge will help for early screening of cancer and promote risk reduction	Yes	495	87.6
	No	70	12.4
Whether telemedicine advice will improve screening and treatment	No	191	33.8
	Yes	374	66.2
Whether you think cancer is a public health concern in Arunachal Pradesh	Highly	420	74.3
	Moderate	115	20.4
	Not a concern	30	5.3
Whether cancer prevention education should be provided at all schools at the college/university level	No	21	3.7
	Yes	544	96.3
Nature of attitude	Negative	84	14.9
	Positive	481	85.1

**Table 7.** Family history of cancer among the participants (*N* = 565).

Parameter	Category	*n*	%
Status of family history of cancer	Death occurred	72	12.7
	No history	468	82.8
	Treated successfully	9	1.6
	Undergoing treatment	16	2.8
Place of screening of family member for the first time (*n* = 97)	Government hospital	44	45.4
	Private hospital	53	54.6
Place of treatment (*n* = 97)	In Arunachal Pradesh	27	27.8
	Outside state	44	45.4
	Partially at both places	26	26.8
Acquisition of medicine for family member’s cancer treatment (*n* = 97)	Partially government and partially private	45	46.4
	Govt. free medicine from other sources	12	12.4
	Purchased from private shop	40	41.2

**Table 8.** Financial implications of cancer treatment (*N* = 565).

Parameter	Category	*n*	%
Cancer treatment expenditures of a family member (*n* = 97)	Above 10 Lakh	9	9.3
	Do not want to mention	29	29.9
	Rs 1 Lakh–Rs 5 Lakh	23	23.7
	Rs 10,000–Rs 50,000	6	6.2
	Rs 5 Lakh–Rs 10 Lakh	22	22.7
	Rs 50,000–Rs 1 Lakh	8	8.2
Socioeconomic impact on the family owing to the cancer treatment of family member (*n* = 97)	Job loss	8	8.2
	Single source of income lost	30	30.9
	Any other	28	28.9
	Do not want to mention	31	40.0
Whether received any financial assistance under any insurance scheme/govt. scheme (n = 97)	No	81	83.5
	Yes	16	16.5
Financial assistance sources (*n* = 16)	Chief Minister Insurance Scheme	12	75.0
	Special cancer package from the government	4	25.0
Was the assistance sufficient (*n* = 16)	Yes	4	25.0
	No	12	75.0
Additional financial sources availed (*n* = 16; multiple responses available)	Personal finances	14	87.5
	Borrowed from relatives	4	25.0
	Sold property and jewelry	5	31.2
	Others	4	25.0

**Table 9.** Factors affecting knowledge about cancer among participants (*N* = 565).

Factor	Category	Knowledge level						*p*
		Poor (*n* = 134)		Moderate (*n* = 234)		Good (*n* = 197)		
		*n*	%	*n*	%	*n*	%	
Age (years)	18 to 24	53	39.6	92	39.3	92	46.7	**0.006**
	25 to 34	45	33.6	109	46.6	74	37.6	
	35 or older	36	26.9	33	14.1	31	15.7	
Sex	Women	78	58.2	115	49.1	108	54.8	0.212
	Men	56	41.8	119	50.9	89	45.2	
Education	Up to higher secondary	68	50.7	60	25.6	45	22.8	**<0.01**
	Graduate	42	31.3	142	60.7	126	64.0	
	Post-graduate and above	24	17.9	32	13.7	26	13.2	
Marital status	Single	84	62.7	192	82.1	158	80.2	**<0.01**
	Married	50	37.3	42	17.9	39	19.8	
Religion	Buddhist	27	20.1	48	20.5	26	13.2	**0.032**
	Christian	72	53.7	99	42.3	93	47.2	
	Hindu and others	35	26.1	87	37.2	78	39.6	
Social caste	SC/ST	128	95.5	216	92.3	186	94.4	0.425
	Non-SC/ST	6	4.5	18	7.7	11	5.6	
Occupation	Daily wage labourer/self-employed	24	17.9	45	19.2	23	11.7	0.167
	Govt/non-govt employee	27	20.1	58	24.8	45	22.8	
	Non-working (homemaker/retired/unemployed/student)	83	61.9	131	56.0	129	65.5	
Community	Arunachali	125	93.3	212	90.6	183	92.9	0.565
	Non-Arunachali	9	6.7	22	9.4	14	7.1	
Type of ration card	APL	19	14.2	26	11.1	30	15.2	0.269
	BPL	35	26.1	44	18.8	40	20.3	
	Do not have	80	59.7	164	70.1	127	64.5	
Type of health insurance availed	Govt/private	11	8.2	21	9.0	24	12.2	0.406
	No insurance	123	91.8	213	91.0	173	87.8	
Participated in other NCDs screening programme	No	119	88.8	213	91.0	167	84.8	0.129
	Yes	15	11.2	21	9.0	30	15.2	
Number of cancer information sources	Up to two sources	70	52.2	37	15.8	32	16.2	**<0.01**
	Three to four sources	56	41.8	153	65.4	94	47.7	
	More than four sources	8	6.0	44	18.8	71	36.0	
Number of known causes of cancer	Up to four causes	121	90.3	62	26.5	1	.5	**<0.01**
	Five–eight causes	13	9.7	171	73.1	145	73.6	
	More than eight causes	0	0.0	1	0.4	51	25.9	
Number of known symptoms of cancer	Up to three symptoms	130	97.0	126	53.8	22	11.2	**<0.01**
	More than three symptoms	4	3.0	108	46.2	175	88.8	
Number of preventive measures practiced	Up to three	101	75.4	48	20.5	7	3.6	**<0.01**
	Four–six	31	23.1	161	68.8	84	42.6	
	More than six	2	1.5	25	10.7	106	53.8	

P-values < .05 were deemed significant

**Table 10.** Factors affecting attitude about risk factors of cancer among the participants (*N* = 565).

Factor	Category	Attitude				*p*
		Negative (*n* = 84)		Positive (*n* = 481)		
		*n*	%	*n*	%	
Age (years)	18 to 24	33	39.3	204	42.4	0.160
	25 to 34	30	35.7	198	41.2	
	35 years or older	21	25.0	79	16.4	
Sex	Women	32	38.1	269	55.9	**0.003**
	Men	52	61.9	212	44.1	
Education	Up to higher secondary	33	39.3	140	29.1	0.126
	Graduate	38	45.2	272	56.5	
	Post-graduate and above	13	15.5	69	14.3	
Marital status	Single	59	70.2	375	78.0	0.122
	Married	25	29.8	106	22.0	
Religion	Buddhist	17	20.2	84	17.5	0.829
	Christian	38	45.2	226	47.0	
	Hindu and others	29	34.5	171	35.6	
Social caste	SC/ST	77	91.7	453	94.2	0.378
	Non-SC/ST	7	8.3	28	5.8	
Community	Arunachali	74	88.1	446	92.7	0.148
	Non-Arunachali	10	11.9	35	7.3	
Occupation	Daily wage labourer/self-employed	15	17.9	77	16.0	0.326
	Govt/non-govt employee	14	16.7	116	24.1	
	Non-working (homemaker/retired/unemployed/student)	55	65.5	288	59.9	
Type of ration card availed	APL	14		61	12.7	0.126
	BPL	23	27.4	96	20.0	
	Do not have	47	56.0	324	67.4	
Type of health insurance availed	Govt/private	10	11.9	46	9.6	0.508
	No insurance	74	88.1	435	90.4	
Participated in other NCDs screening programme	No	77	91.7	422	87.7	0.300
	Yes	7	8.3	59	12.3	
Number of sources of information about cancer	Up to two sources	29	34.5	110	22.9	0.062
	Three to four sources	41	48.8	262	54.5	
	More than four sources	14	16.7	109	22.7	
Number of known causes of cancer	Up to four causes	43	51.2	141	29.3	**<0.01**
	Five–eight causes	31	36.9	298	62.0	
	More than nine causes	10	11.9	42	8.7	
Number of known symptoms of cancer	Up to three symptoms	59	70.2	219	45.5	**<0.01**
	More than three symptoms	25	29.8	262	54.5	
Number of preventive measures practiced	Up to three	39	46.4	117	24.3	**<0.01**
	Four–six	24	28.6	252	52.4	
	More than six	21	25.0	112	23.3	

P-values < .05 were deemed significant

**Table 11.** Factors affecting practices against risk factors of cancer among the participants (*N* = 565).

Factor	Category	Level of practice						*p*
		Low (*n* = 177)		Moderate (*n* = 191)		High (*n* = 197)		
		*n*	%	*n*	%	*n*	%	
Age (years)	18 to 24	69	39.0	74	38.7	94	47.7	**0.002**
	25 to 34	61	34.5	89	46.6	78	39.6	
	35 years or older	47	26.6	28	14.7	25	12.7	
Sex	Women	93	52.5	102	53.4	106	53.8	0.970
	Men	84	47.5	89	46.6	91	46.2	
Education	Up to higher secondary	80	45.2	55	28.8	38	19.3	**<0.01**
	Graduate	70	39.5	117	61.3	123	62.4	
	Post-graduate and above	27	15.3	19	9.9	36	18.3	
Marital status	Single	114	64.4	156	81.7	164	83.2	**<0.01**
	Married	63	35.6	35	18.3	33	16.8	
Religion	Buddhist	33	18.6	44	23.0	24	12.2	**0.009**
	Christian	91	51.4	86	45.0	87	44.2	
	Hindu and others	53	29.9	61	31.9	86	43.7	
Social caste	SC/ST	166	93.8	178	93.2	186	94.4	0.883
	Non-SC/ST	11	6.2	13	6.8	11	5.6	
Community	Arunachali	162	91.5	175	91.6	183	92.9	0.859
	Non-Arunachali	15	8.5	16	8.4	14	7.1	
Employment	Daily wage labourer/self-employed	35	19.8	33	17.3	24	12.2	0.146
	Govt/non-govt employee	39	22.0	50	26.2	41	20.8	
	Non-working (homemaker/retired/unemployed/student)	103	58.2	108	56.5	132	67.0	
Type of ration card	APL	16	9.0	23	12.0	36	18.3	**0.004**
	BPL	45	25.4	28	14.7	46	23.4	
	Do not have	116	65.5	140	73.3	115	58.4	
Type of health insurance availed	Govt/private	16	9.0	12	6.3	28	14.2	**0.029**
	No insurance	161	91.0	179	93.7	169	85.8	
Participated in other NCDs screening programme	No	155	87.6	179	93.7	165	83.8	**0.009**
	Yes	22	12.4	12	6.3	32	16.2	
Number of sources of information about cancer	Up to two sources	71	40.1	32	16.8	36	18.3	**<0.01**
	Three to four sources	90	50.8	111	58.1	102	51.8	
	More than four sources	16	9.0	48	25.1	59	29.9	
Number of causes of cancer known	Up to four causes	128	72.3	47	24.6	9	4.6	**<0.01**
	Five–eight causes	45	25.4	140	73.3	144	73.1	
	More than nine causes	4	2.3	4	2.1	44	22.3	
Number of symptoms of cancer known	Up to three symptoms	148	83.6	89	46.6	41	20.8	**<0.01**
	More than three symptoms	29	16.4	102	53.4	156	79.2	
Knowledge on if vaccines have role to prevent certain cancers	No	161	91.0	173	90.6	148	75.1	**<0.01**
	Yes	16	9.0	18	9.4	49	24.9	
Knowledge about NCD patients being at high risk during the COVID-19 pandemic	No	106	59.9	132	69.1	83	42.1	**<0.01**
	Yes	71	40.1	59	30.9	114	57.9	
Knowledge level	Poor	107	60.5	24	12.6	3	1.5	**<0.01**
	Moderate	62	35.0	124	64.9	48	24.4	
	Good	8	4.5	43	22.5	146	74.1	
Attitude	Negative	42	23.7	18	9.4	24	12.2	**<0.01**
	Positive	135	76.3	173	90.6	173	87.8	

P-values < .05 were deemed significant
